# Tumor organoids: applications in cancer modeling and potentials in precision medicine

**DOI:** 10.1186/s13045-022-01278-4

**Published:** 2022-05-12

**Authors:** Hanxiao Xu, Dechao Jiao, Aiguo Liu, Kongming Wu

**Affiliations:** 1 Department of Pediatrics, Tongji Hospital of Tongji Medical College, Huazhong University of Science and Technology, Wuhan, 430030 China; 2 Department of Interventional Radiology, The First Affiliated Hospital of Zhengzhou University, Zhengzhou, 450052 China; 3 Department of Oncology, Tongji Hospital of Tongji Medical College, Huazhong University of Science and Technology, Wuhan, 430030 China

**Keywords:** Organoid, Patient-derived xenografts, Cancer, Tumor microenvironment, Therapy response prediction, Drug discovery

## Abstract

Cancer is a top-ranked life-threatening disease with intratumor heterogeneity. Tumor heterogeneity is associated with metastasis, relapse, and therapy resistance. These factors contribute to treatment failure and an unfavorable prognosis. Personalized tumor models faithfully capturing the tumor heterogeneity of individual patients are urgently needed for precision medicine. Advances in stem cell culture have given rise to powerful organoid technology for the generation of in vitro three-dimensional tissues that have been shown to more accurately recapitulate the structures, specific functions, molecular characteristics, genomic alterations, expression profiles, and tumor microenvironment of primary tumors. Tumoroids in vitro serve as an important component of the pipeline for the discovery of potential therapeutic targets and the identification of novel compounds. In this review, we will summarize recent advances in tumoroid cultures as an excellent tool for accurate cancer modeling. Additionally, vascularization and immune microenvironment modeling based on organoid technology will also be described. Furthermore, we will summarize the great potential of tumor organoids in predicting the therapeutic response, investigating resistance-related mechanisms, optimizing treatment strategies, and exploring potential therapies. In addition, the bottlenecks and challenges of current tumoroids will also be discussed in this review.

## Background

Preclinical tumor models serve as a prominent platform for mechanistic research and testing new drugs. Over the past few decades, clinical trials have witnessed the most failures of novel therapies, despite great efforts in target validation and drug optimization based on conventional preclinical models [1], including cell culture, cell-line or patient-derived xenograft models, and murine or nonmurine animal models [2].

Cancer cells, either growing in culture medium or as xenografts, do not accurately recapitulate the complexity of human cancers due to the deficiency of tumor-initiating cells and the absence of a human-specific tumor microenvironment (TME) and extracellular matrix (ECM) [3], as well as the genetic variance resulting from long-term maintenance and passages in vitro [4]. For patient-derived xenograft (PDX) models, sample accessibility, logistic and economic issues, and ethical concerns hamper their broad and extensive application in basic research and personalized medicine [5].

**Fig. 1:**
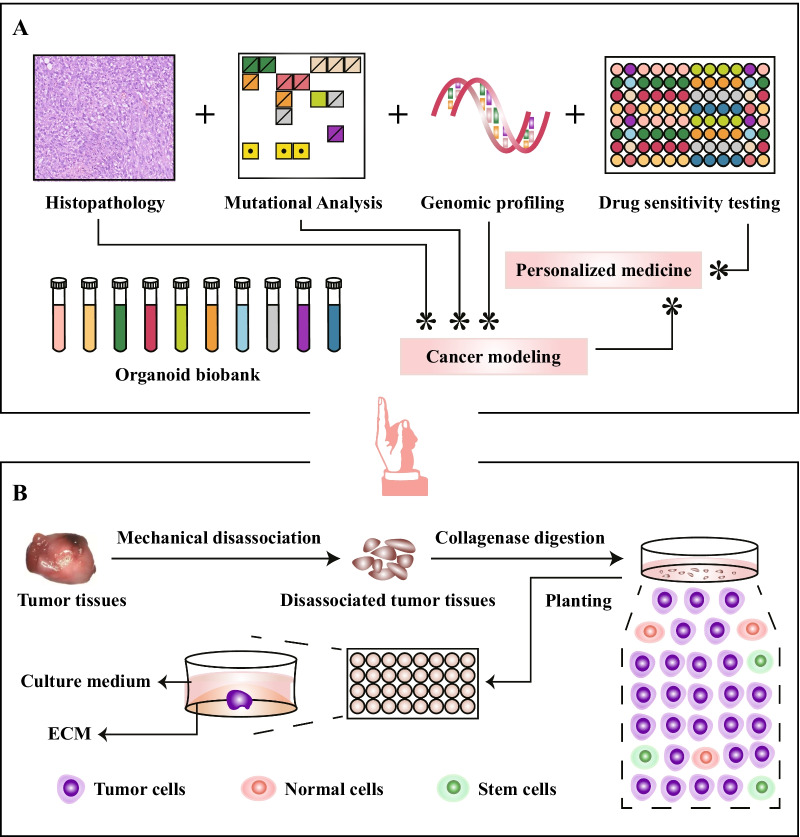
Main steps of PDO generation and main applications of PDOs. Human cancer tissues containing cancer cells, adult stem cells, pluripotent stem cells, or cancer stem cells are occasionally first disassociated into very small pieces, cell clusters, or single cells using mechanical and chemical methods and cultured under proper 3D conditions in hydrogels with ECM components (**A**). Tumoroids mimic the primary tissues in terms of histopathological features, genetic profiles, mutational landscape, and even responses to therapy, and tumoroid biobanks can be established (**B**). 3D, three-dimensional; ECM, extracellular matrix; PDO, patient-derived organoid

Organoids are in vitro tissues that originate from human stem cells, organ-specific progenitor cells, or even disassociated tumor tissues and are cultured in proper ECM-based medium with relatively high success rates. Tumoroids mimic the primary tissues in both architecture and function and retain the histopathological features, genetic profile, mutational landscape, and even responses to therapy (Fig. 1A) [6, 7]. Thus, organoids serve as excellent tools for investigating tumorigenesis and cancer progression in vitro and exhibit enormous potential for translational studies [6–15]. To date, organoids derived from multiple human tumor types have been successfully established [13, 16–19]. Compared with PDX models, organoid establishment requires less time and less tissue, and tumoroids stably maintain the key characteristics of primary tumors even after long-term passaging [13]. Furthermore, in vitro gene modification is much easier than in vivo, as exemplified by genetically manipulated organoids with genetic knockouts of the tumor suppressors *Trp53* and *Stag2* via CRISPR/Cas9 technology [18]. In this review, we summarize and outline recent progress in organoid technology in preclinical and clinical cancer research.

## Organoid culture system

The processes used to generate distinct patient-derived organoids (PDOs) are different to varying degrees but generally share several main steps. Human tumor tissues containing pluripotent stem cells (PSCs), adult stem cells, or cancer stem cells are occasionally first disassociated into very small pieces, cell clusters, or single cells using mechanical and chemical methods and then cultivated under proper culture conditions in hydrogels with ECM components, among which Matrigel is commonly utilized (Fig. 1B). These culture systems maintain cell proliferation and differentiation, including stem cells [20], and these cells self-organize into functional units or specific tissue architectures that contain both differentiated cells and stem cells [20, 21].

Sample processing methods mainly include mechanical disassociation and chemical digestion. Yawei Hu et al. reported an adapted mechanical processing method for cancer tissues that promotes the efficient generation of lung cancer organoids (LCOs) in 3 days [22]. This sample processing method reached a 79% success rate, which was further enhanced by removing tumor tissues exhibiting serious necrosis, fibrosis, and carbon deposition [22]. Compared with conventional enzyme digestions, mechanical processes generated more tumoroids, possibly due to the maintenance of intercellular connections within cell clusters during mechanical processing [22]. In contrast, enzyme digestion increased the amount of normal lung tissue-derived spheroids obtained, probably because enzyme treatment contributed to the release of epithelial cells from normal lung tissues [22].

Some growth factors and inhibitors are required in the culture medium (Table 1). Different combinations of growth factors and inhibitors in the medium contribute to the generation of distinct component lineages in organoids [23]. For instance, a recent study revealed that the removal of epidermal growth factor (EGF) contributes to a relatively higher proportion of mature luminal cells with downregulation of mucin-1 and galectin-1, as well as a lower proportion of basal cells with upregulation of cluster of differentiation 90 (CD90) [23]. In addition, the elimination of heregulin-*β*1, p38 mitogen-activated protein kinase (MAPK) inhibitor, or fibroblast growth factor 7 (FGF7) and FGF10 also exerts significant effects on the distribution of mammary lineages, such as mature luminal cells and luminal progenitor cells. In addition, B27 removal contributes to reducing the number of basal cells expressing CD73/CD90 [23].

**Table 1:** Tumoroid culture system of common cancer types

Tumor type	Origin type	Culture system	Application	References
Lung cancer (Five subtypes of lung cancer)	Surgically resection	Matrigel MBM which consists of serum-free DMEM/F12 medium supplemented with bFGF, human EGF, N2, B27, ROCK inhibitor, and 1% penicillin/streptomycin	Tumor modeling in histology, genetic characteristics, PD-L1 expression Drug sensitivity testing	[16]
NSCLC (Adenocarcinomas and squamous cell carcinomas)	Surgically resected primary tumors with early-stage NSCLC NSCLC PDX tissues	100% growth factor-reduced Matrigel Advanced DMEM/F12 supplemented with GlutaMAX, HEPES, Antibiotic–Antimycotic, B27, N-Acetylcystenine; recombinant human EGF, FGF-10, FGF-4, and Noggin; A83-01, Y-27632, SAG, CHIR 99021	NSCLC modeling in histology and genetic profiling Drug response testing: the response of KRAS-mutant organoids to MEK inhibitors The response of FGFR-amplified organoids to the combination of trametinib and BKM120 with BGJ398	[24]
Breast cancer (Major disease subtypes: triple-negative, ER-positive/PR-positive, Her2-positive)	Surgical resection	BME Type 1: Advanced DMEM/F12 supplemented with R-spondin 1, Noggin, B27, Vit A, Nicotinamide, N-Acetylcystenine, Primocin, Y-27632 (optional), Heregulin-B1, FGF-7, FGF-10, A83-01, EGF, SB202190 Type 2: The addition of Wnt3a, Hydrocortisone, beta-estradiol, and Forskolin, and the removal of FGF-7 and SB202190 on the basis of Type 1	Genetic manipulation in vitro Orthotopic organoid transplantation in mice with estrogen pellets	[63]
Gastric cancer	Gastrectomy specimen	Matrigel Advanced DMEM/F12, GlutaMax, HEPES, penicillin/streptomycin, Wnt3a, R-spondin1, Noggin, B27, EGF, FGF10, N-Acetylcystenine, Gastrin, A83-01, Y-27632, primocin	Gastric cancer modeling; recapitulation of genomic and transcriptomic features High-throughput drug screen Discovery of potential target drugs	[68]
Liver cancer (HCC, intrahepatic cholangiocellular carcinoma)	Needle biopsies	Reduced growth factor BME2 Advanced DMEM/F-12 supplemented with B-27, N-2, Nicotinamide, N-Acetylcystenine, gastrin, forskolin, A83-01, EGF, FGF10, HGF, R-spondin1, Wnt3a Adapted medium with addition of FGF19 as well as lack of forskolin, HGF, N-Acetylcystenine, and Nicotinamide	Liver cancer modeling Recapitulation of histopathological characteristics, somatic genetic alterations Drug sensitivity testing HCC and intrahepatic CCC organoids show differential sensitivity to sorafenib	[84]
PDAC	Pancreatectomy	Growth factor-reduced Matrigel Advanced DMEM/F12 medium supplemented with HEPES, GlutaMax, penicillin/streptomycin, B27, N-Acetylcystenine Wnt-3a, R-spondin1, Noggin, EGF, FGF, Nicotinamide, Y-27263 and A83–01	Investigation in tumor metastasis-related mechanism Inhibition of ERK1/2 in cancer-associated pancreatic stellate cells suppresses cancer–stromal interaction and metastasis	[102]
Colorectal cancer	Biopsy	Matrigel Advanced DMEM/F12 medium supplemented with R-spondin 1, Noggin, EGF, HEPES, Glutamax, Normocin, Gentamicin/Amphotericin B, N2, B27, N-Acetylcysteine, Niacinamide, A83-01, SB202190, Gastrin, Prostaglandin E2	Gastric cancer modeling; recapitulation of histopathology and genomic characteristics Drug sensitivity testing: Irradiation, 5-Fluorouracil, and Irinotecan	[112]
Renal cancer	Surgical resection	Collagen I Advanced MEM/F12 supplemented with Wnt3a, R-spondin 1, HEPES, Glutamax, Nicotinamide, N-Acetylcysteine, B27 without VitA, A83-01, SB-202190, Penicillin/Streptomycin, Gastrin, EGF, Noggin, Normocin, IL-2	Gastric cancer modeling Recapitulation of histopathology Presence of cytotoxic immune cells Differential response to drugs	[118]
Bladder cancer	Cystectomy	BME Advanced DMEM/F-12, FGF10, FGF7, FGF2, B27, A83-01, N-Acetylcysteine, Nicotinamide (10 mM) Y-27632 is added after passaging	Gastric cancer modeling Therapy response prediction	[18]
Prostate cancer	Prostate needle biopsy Transurethral resection Prostatectomy Circulating tumor cells	ECM Advanced DMEM/F12 medium supplemented with Penicillin/Streptomycin, HEPES, GlutaMAX, B27, Nicotinamide, N-Acetylcysteine, EGF, A83-01, Noggin, R-spondin 1, Dihydrotestosterone, FGF2, FGF10, Prostaglandin E2, SB202190 Y-27632 is added after passaging	Tumor modeling	[133]

BME2, basement membrane extract, Type 2; ECM, extracellular matrix; EGF, epidermal growth factor; ER, estrogen receptor; FGF, fibroblast growth factor; HER2, human epidermal growth receptor 2; HGF, hepatocyte growth factor; MBM, minimum basal medium; NSCLC, non-small cell lung cancer; PDAC, pancreatic ductal adenocarcinoma; PDO, patient-derived organoid; PR, progesterone receptor

The outgrowth of normal tissues, which reduces the purity of cancer organoids, is a common and challenging issue for increasing the purity of tumoroids [24], as exemplified by the study conducted by Krijn K Dijkstra et al., showing that the success rate of pure non-small cell lung cancer (NSCLC) organoid establishment was only 17% [25]. Minimizing the growth of normal tissues during the establishment and maintenance of tumor organoids is important and challenging. Culture medium components exert different effects on the expansion of normal and cancer tissues [26]. Only complete medium supports the long-term growth of normal gastric organoids [26]. For gastric cancer organoids (GCO), the absence of Wnt, A38-01, and FGF10 does not affect the phenotypes [26]. According to the detailed protocol reported by Kim M and colleagues, 30% Wnt3A-conditioned medium, Noggin, and A83-01 were added to normal bronchial organoids compared with the culture medium of LCOs [16]. Thus, in addition to a more accurate extraction of tissue materials and an appropriate sample processing approach, eliminating medium components that are essential for normal organoid growth but do not affect tumoroid growth might be a possible candidate solution for this issue.

## Human tumor modeling

### Lung cancer

Lung cancer (LC) represents the most common tumor type and causes most cancer-related mortality worldwide [27]. To date, accumulating evidence has revealed the feasibility and superiority of LCOs [16, 22, 24, 25, 28–44]. Kim M et al. reported the successful establishment of organoids representative of five LC subtypes [16]. These LCOs sufficiently retained the morphological and histological features and the genomic variations of the corresponding primary LC during long-term passage in vitro [16]. For instance, acinar or large glandular structures were observed in lung adenocarcinoma organoids along with the expression of tumor markers, including napsin-A and cytokeratin 7 (CK7) [16]. Squamous cell LCOs displayed some histologic features of this tumor subtype, such as different cell borders, cytoplasmic keratinization, and high expression of some squamous cell carcinoma-specific markers (p63 and CK5/6) [16]. At the genomic level, a high level of consistency was observed in single-nucleotide polymorphism profiles and somatic nonsynonymous mutations among 44 cancer-related genes between LCOs and matched original cancer tissues [16]. Driver gene mutations, including *TP53* and *epidermal growth factor receptor (EGFR)*, in their original tumors were also recapitulated in LCOs [16].

Much effort has been exerted to model tumorigenesis and progression, as well as to further elucidate the molecular mechanisms using tumor organoids [29, 30]. Dost AFM et al. utilized organoid cultures to investigate the early molecular events occurring in lung epithelial cells following oncogenic *KRAS* activation [38]. Decreased expression of mature lineage identity genes and upregulation of developmental and progenitor genes were detected in alveolar epithelial progenitor cells harboring oncogenic *KRAS* [38]. Another study based on tumor organoids and genetically engineered mouse models showed that phase separation of EML4-ALK signaling triggered LC formation and malignant transformation [29]. In addition, human-induced PSC (iPSC)-derived lung organoids with human epidermal growth receptor 2 (HER2) overexpression contained atypical adenomatous hyperplasia-like structures with a higher degree of proliferation and morphological abnormalities, as well as the activation of oncogenic signaling pathways, including RAS/RAF/MAPK and phosphatidylinositol 3-kinase (PI3K)/protein kinase B (AKT)/mammalian target of rapamycin (mTOR) signaling [30]. This phenomenon indicates that HER2 also drives the carcinogenesis of lung adenocarcinoma. In addition, cyclin-dependent kinase 1 (CDK1), CCNB2, and CDC25A also exert tumor-promoting effects on the tumorigenesis and development of lung adenocarcinoma organoids [31].

### Breast cancer

Breast cancer (BC) represents the most common malignant disease in females and displays high heterogeneity [45, 46]. The differential expression profile of pivotal signaling pathways and molecules, including estrogen receptor (ER), progesterone receptor (PR), and HER2, plays important roles in cancer biological behaviors, affects the choices of therapeutic intervention, and is associated with clinical outcomes [47, 48]. To date, a large repository of research has reported the successful development of breast cancer organoids (BCOs) [13, 23, 49–63]. In a previous study conducted by Sachs N et al., organoids were generated from primary and metastatic breast cancer tissues and accurately reproduced the histopathology, hormone receptor status, HER2 status, and DNA copy number variations [13]. Fang G et al. reported the high-throughput establishment of mouse mammary tumoroids using nonadhesive alginate instead of basement membrane extract (BME) through microfluidic droplet technology [64]. These mammary tumoroids contained luminal and solid-like architectures and showed similar cellular phenotypes and lineages to the original tumors [64]. Rosenbluth JM and colleagues generated 79 organoids derived from normal breast tissues and high-risk tissues from patients with breast carcinoma [23]. These high-risk tumoroids reproduced the propagation of BRCA1 heterozygosity-related luminal progenitor cells [23].

Integration of CRISPR/Cas9 technology and human BCOs has also been conducted to investigate the onset of BC [51]. Genetic knockout of tumor suppressor genes, including *P53*, *phosphate and tension homology deleted on chromosome ten*, *RB1*, and *neurofibromatosis type-1,* endowed organoids with a long-term culture capacity [51]. Another study revealed that *SOX4* promotes the maintenance of undifferentiated and proliferative states in mammary tumoroids [62]. Compared with *SOX4*-proficient tumoroids, tumoroids with *SOX4* knockout contained more differentiated cells with luminal or basal gene expression patterns and lower levels of cell cycle genes and showed an impaired capacity for tumor growth and metastatic outgrowth [62].

### Gastric cancer

Gastric cancer is a common malignancy worldwide with marked molecular heterogeneity and is the second leading cause of cancer-related deaths [65, 66]. Many studies have reported the successful generation of GCOs [12, 26, 67–80]. Seidlitz T documented the feasibility of establishing a gastric cancer organoid biobank from human gastric or esophagogastric adenocarcinoma and mouse gastric cancer tissues [26]. These organoids retained the divergent growth behavior and morphological phenotypes, as well as histological characteristics of the respective parent tissues to a great extent [26]. The morphological and histological characteristics remained unchanged even after long-term culture for 1 year [26]. For instance, corpus carcinoma-derived organoids harbored a cystic structure containing a thickened epithelium, and antrum carcinoma-derived organoids did not contain a lumen [26]. The expression of distinct gastric cancer markers, including CK7, cadherin-17, and carcinoembryonic antigen, and the periodic acid–Schiff staining pattern in original cancer tissues were also faithfully and permanently maintained in gastric tumoroids [26]. In addition, a mutational spectrum was revealed among these tumoroids using whole-genome sequencing that was consistent with the previously reported genomic stable (GS) subtype, microsatellite instable (MSI) subtype, and chromosomal instable (CIN) subtype of gastric cancer [26]. In another study, gastric tumoroids, including the subtypes of EBV, MSI, CIN, and GS, as well as *CLDN18-ARHGAP6* or *CTNND1-ARHGAP26* fusions or mutations in *RHOA*, also faithfully recapitulated architectural and regional heterogeneity, as well as the morphology and transcriptomic and genomic profiles, even after long-term culture [68].

### Liver cancer

Liver cancer, which is classified into hepatocellular carcinoma (HCC), intrahepatic cholangiocarcinoma, and mixed type, is among the most common digestive tract tumors with an increased incidence, unfavorable clinical outcomes, and high mortality [81]. Liver cancer organoids have been generated in an increasing number of studies to better model this tumor type [82–92]. According to one previous study, liver cancer organoids derived from needle biopsy tissues faithfully model the histopathological features of matched patient tumors even after long-term culture for up to 1 year [84], including the growth pattern, differentiation grade, and the expression patterns of the tumor marker alpha-fetoprotein, glypican 3, glutamine synthetase, and heat shock protein 70 [84].

Fibroblast growth factor receptor 2 (FGFR2) fusion proteins were reported to promote oncogenic transformation of mouse liver organoids to cholangiocarcinoma [93]. In another study, functional human HCC organoids were generated from Huh7 cells, human iPSC-derived mesenchymal cells (MCs), and human iPSC-derived endothelial cells (ECs) [94]. The combination of human iPSC-ECs and iPSC-MCs drove HCC growth, and the immune response was important for slowing tumor growth at an early stage [94]. Sequential knockout and knock-in of driver mutations using CRISPR–Cas9 technology were conducted to generate genetically modified liver cancer organoids as a method to further investigate tumor pathogenesis [95]. These observations indicate the great potential of liver cancer organoids in tumor modeling and subsequently providing a better understanding of liver cancer biology.

### Pancreatic cancer

Pancreatic cancer represents one of the most lethal solid tumor types; pancreatic ductal adenocarcinoma accounts for 90% of these tumors, and less than 5% are pancreatic neuroendocrine neoplasms [96, 97]. Pancreatic cancer organoids have been generated to investigate tumor biology and promote clinical applications [96, 98–103]. PDOs derived from primary pancreatic ductal adenocarcinoma (PDAC) and matched liver metastases were generated [104]. A single-cell analysis was subsequently performed using these tumoroids. These organoids contained both “classical” cells and “basal-like” cells and showed two different cell states of a cycling progenitor cell and a differentiated secretory connected through a differentiation hierarchy [104]. A functional hierarchy of PDAC cell states was correlated with transcriptional patterns in tumor subtypes, supporting the potential application of organoids as a coclinical model in studies of cancer heterogeneity [104, 105]. In addition, pancreatic cancer PDX-derived organoids were shown to retain complex glycosylation variations [4] that contribute to cancer development by dysregulating protein expression levels, stability, and localization [106]. KRAS was shown to regulate epithelium–macrophage cross talk and promote pancreatic carcinogenesis in a pancreatic organoid model [103]. Coculture models suggested that heterospheroids consisting of primary human PDAC cells and pancreatic stellate cells were more resistant to gemcitabine than PDAC-only spheroids, and a further mechanistic study showed that deoxycytidine secreted from pancreatic stellate cells mediated PDAC resistance to gemcitabine [105].

### Colorectal cancer

Accumulating studies have employed colorectal cancer (CRC) organoids to investigate tumor development [90, 107–113]. Organoids derived from metastatic CRC and metastatic gastroesophageal cancer show great similarity with the respective biopsies in terms of morphology, the mutational spectrum, genes with an altered copy number, and expression patterns of common clinical diagnosis markers, including caudal-related homeobox 2 (CDX2) and CK7 [12].

Ganesh K and colleagues have also developed a biobank of 65 PDOs of rectal cancer derived from patients with primary, metastatic, or recurrent rectal cancer and achieved a success rate of 77% at a whole level [107]. These tumoroids have been generated even from minute amounts of tumor tissues obtained from endoscopic biopsies [107]. Consistently, rectal cancer organoids faithfully recapitulate the molecular and histopathological characteristics of the matched primary tumors [107]. For instance, some markers expressed in these tumoroids reflect the matched origin parent tumors, which was verified by the detection of CDX2, high expression of E-cadherin, β-catenin staining patterns in the cytoplasm and nucleus, or goblet cells with mucin-2 expression, similar to the primary tumors [107]. Additionally, these tumoroids maintained specific glandular features in terms of architecture (e.g., cord- and nest-like growth patterns, nuclear stratification, and pooled mucin production) and subtle cytologic features (e.g., cytoplasmic clearing and cytoplasmic eosinophilia) [107]. In addition, tumoroids also retain the mutational fingerprint of the matched primary tumors with approximately 92% concordance [107].

Another study documented the feasibility of tumoroids in the accurate recapitulation of *KRAS*-mutant metastatic rectal cancer with microsatellite stability after hepatic resection and treatment with neoadjuvant combination chemotherapies of 5-FU, leucovorin, and oxaliplatin [114]. The histopathological differentiation phenotypes of these PDOs were consistent with liver metastases [114]. Collectively, colorectal tumoroids accurately model native tumors and facilitate mechanistic research on this cancer type.

### Renal cancer

Renal cell carcinoma (RCC) is a common urinary system malignancy associated with high cancer-related mortality [115, 116]. Many studies have reported the successful generation of renal cancer organoids for accurate cancer modeling [117–119]. Patient RCC tissue and matched tumoroids shared high similarity in histopathological characteristics, including chromophobe RCC and sarcomatoid variant renal carcinoma [117]. In addition, these tumoroids retain some genetic features of native tumors, as exemplified by consistency in numerous gene mutations, such as mutations in von Hippel–Lindau and polybromo 1 [117]. Using organoids derived from patients with RCC, Hamdan F et al. reported that an oncolytic adenovirus secreting the cross-hybrid Fc-fusion peptide that binds to programmed death-ligand 1 (PD-L1) and activates the Fc-effector significantly increases tumor killing and minimizes toxicity compared with the PD-L1 inhibitor atezolizumab, IgG1-PDL1 and IgA-PDL1 [119].

### Bladder cancer

Bladder cancer is a common urothelial carcinoma that seriously affects the survival and quality of life of patients. Much effort has been made to develop an efficient long-term culture system for bladder cancer organoids [18, 120]. According to a previous study conducted by Mullenders J and colleagues, bladder cancer organoids are successfully established from surgically resected tumors and biopsies with approximately 50% efficiency and are maintained and passaged long term [18]. Bladder cancer organoids faithfully and accurately recapitulate the respective primary tumors in terms of histology and function [18]. Organoids encompass both luminal and basal bladder cancer subtypes and retain common cancer-related mutations, such as *TP53* and *FGFR3* [18]. Consistent with the primary tumors, organoids display distinct morphological structures. For instance, organoids exhibit either solid or lumen-containing and smooth rounded, elongated structures or a very irregular morphology [18].

In another study by Lee SH et al., bladder cancer organoids were developed from transurethral resected samples containing both low-grade nonmuscle invasive tumor and high-grade muscle invasive cancer with approximately 70% efficiency [121]. Consistently, organoids retained the tumor heterogeneity and mutational spectrum of matched primary cancer. Furthermore, these tumoroids displayed a series of genomic alterations that were consistent with cancer evolution in culture [118, 121].

### Prostate cancer

Prostate cancer (PC) represents one of the most common cancer types in males and has an increasing incidence worldwide; it also ranks among the highest level of cancer-related mortality in males [122]. Many studies have employed organoids to investigate tumor biology and promote tumor translational medicine [19, 123–133]. Heninger E and colleagues established PDOs from locally advanced PC [134]. According to orthogonal analyses, these organoids retained the complexity of the TME observed in multifocal PC [134]. According to an orthogonal flow cytometry analysis, PC organoids retained a distinct subpopulation of epithelial cells that conserved the expression of AR-related molecules [134]. Based on genetically normal mouse prostate organoids, *SPOP* mutation contributes to accessibility and AR binding patterns that are similar to those of native primary tumors [135].

## Modeling tumor vascularization

**Fig. 2:**
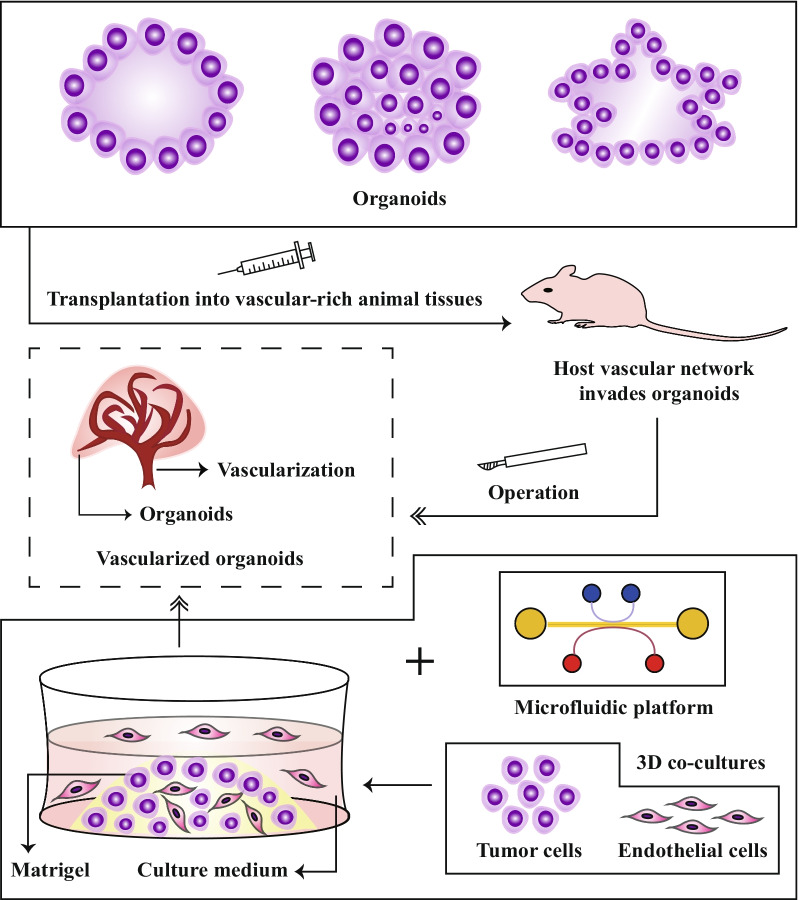
Workflow of organoid vascularization. Implantation of tumoroids into highly vascularized tissues in animals is an effective approach for organoid vascularization. After organoids are engrafted in vasculature-rich mouse tissue, the host vasculature infiltrates the organoids. Another approach to generate vascularized organoids is combining the coculture of mixed cells or microfluidic platforms

A normal vasculature contains ECs, vascular smooth muscle cells, pericytes, and ECM [136, 137]. Angiogenesis plays pivotal roles in tumor growth and metastasis by delivering the required nutrients for growth and acting as a passageway for tumor cells to escape to other habitats [138]. Recently, vascular endothelial growth factor receptor (VEGFR)-associated targeted agents have been proven to be effective antitumor therapies against multiple solid tumors [139]. Much effort has been made to recapitulate the tumor vasculature in organoid cultures for a better understanding of angiogenic signaling pathways and for developing effective treatment strategies [136, 140]. Implantation of organoids into highly vascularized tissues is a commonly applied approach to organoid vascularization [141] (Fig. 2). After organoids are engrafted in the vasculature-rich animal tissue, the host vasculature infiltrates the organoids and connects the host animal model to the transplanted organoid model [142]. Following the implantation of human brain organoids into adult mouse brains, extensive infiltration of the host vasculature was observed [143]. Another approach to vascularize organoids is combining cocultures of mixed cells through gene editing or using a microfluidic platform.

Philipp Wörsdörfer et al. reported that mesodermal progenitor cell-derived tumoroids exhibit typical features of a blood vessel, such as luminal caveolae, EC junctions, and basement membrane [142]. According to another study, the hepatocellular carcinoma organoid system expressed markers of epithelial–mesenchymal transition, neoangiogenesis, and inflammation at relatively high levels, including vimentin, transforming growth factor beta (TGF-β), VEGFR2, hypoxia-inducible factor 1α, VEGF, CXCR4, and tumor necrosis factor α, when cocultured with ECs and fibroblasts [144].

When cultured adjacent with the vascular network, PDMS-based patient-derived BCOs containing ECs and immune cells display a significant angiogenic response for 3 weeks [145]. When cultured near cancer-associated fibroblasts (CAFs), these organoids show significantly increased expression of VEGF-A and TGF-β [145]. In another study, vascularized breast tumoroids were also generated in collagen- and hyaluronic acid-enriched ECM, which contained MCF-7 cells and human fibroblasts [146]. Vascularized tumoroids were also successfully constructed by coculturing tumor cells with EC-derived endothelial colony-forming cells and lung fibroblasts [147]. Based on compartmentalized microfluidic chips, VEGF and hypoxia gradients induced and guided vascularization in the coculture system of organoids and endothelial cells [141, 148]. Generally, coculture strategies promote organoid vascularization.

## Modeling of tumor–immune interactions

**Fig. 3:**
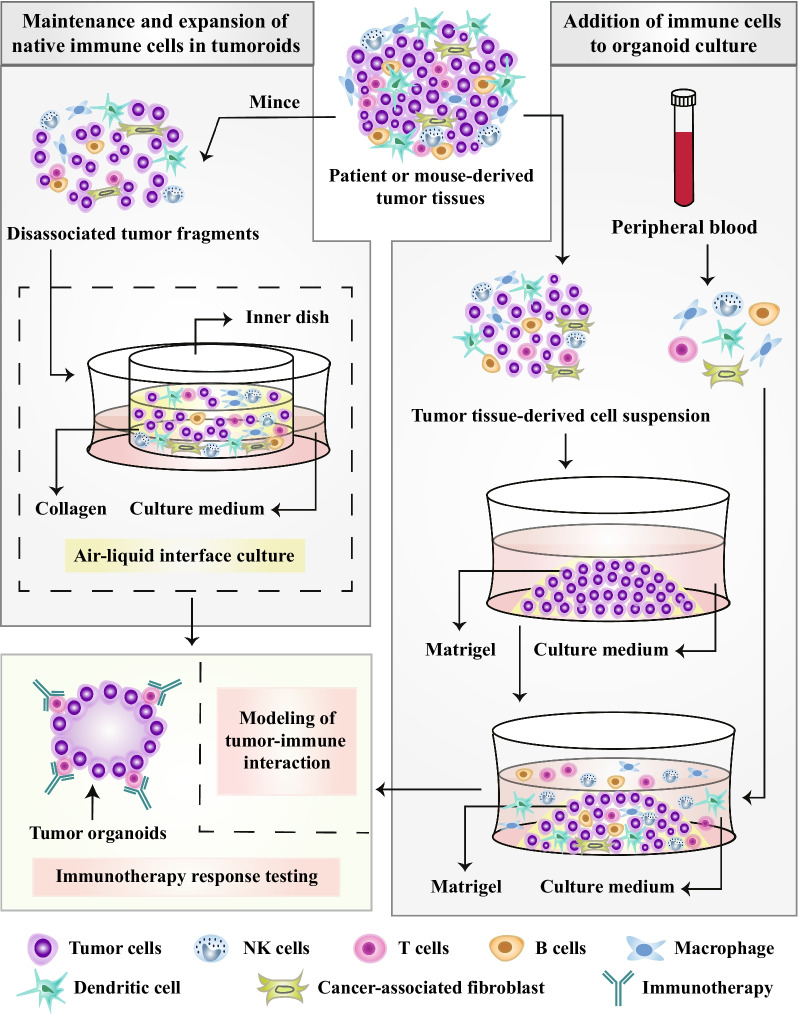
Modeling the immune microenvironment in a coculture system of tumoroids and immune cells. Two approaches have been developed to coculturing organoids and immune cells: maintenance and expansion of native immune cells in tumoroids and addition of immune cells to organoid culture. Immune cells can be obtained from the ALI culture system. Tumoroids are embedded in a collagen gel with one side exposed to air and the other side in contact with the liquid culture medium. Cocultures of tumoroids and immune cells may promote the prediction and evaluation of individual tumor responses to clinically used immunotherapies

Cancer immunotherapy brings new hope for cancer treatment [149–152]. The tumor immune microenvironment affects tumor growth and progression, as well as cancer responses to immunotherapy [153, 154]. Enormous efforts have been made to improve current technological platforms for better modeling immune system function in tumors [155–160]. The coculture of tumoroids and immune cells represents a commonly used and effective approach (Fig. 3). Currently, two approaches to cocultures of organoids and immune cells are used, including maintenance and expansion of native immune cells in tumoroids and addition of immune cells to organoid culture [161]. However, a robust anticancer immune response requires the interaction between tumor tissue-infiltrated innate immune cells and acquired immune cells, such as neutrophils, MDSCs, macrophages, NK cells, DCs, and T cells. In addition, various cytokines are also involved in this process. These cells and cytokines work together to determine the immune response and immunotherapeutic effect. At present, mimicking the real tumor environment with an in vitro system is nearly impossible [162–165].

Cocultures of gastric cancer organoids and immune cells were utilized to study the immunosuppressive function of myeloid-derived suppressor cells (MDSCs), revealing that PD-L1 expression is regulated by the mTOR signaling pathway in gastric cancer [155]. Furthermore, enhanced tumor growth and impaired proliferation of cytotoxic T cells were observed in cocultures of pancreatic cancer organoids with MDSCs [156]. In addition, 3D cocultures of pancreatic cancer cells with monocytes and CAFs were also established, in which increased secretion of immunosuppressive cytokines was detected, and these cytokines inhibited activation and proliferation in vitro [157]. Dijkstra KK and colleagues cocultured autologous cancer organoids and peripheral blood lymphocytes [158]. These cocultures have been shown to be capable of enriching tumor-reactive T cells from patient-derived peripheral blood [158]. Tumor-reactive T cells contributed to a reduced organoid size and widespread apoptosis [158].

The air–liquid interface (ALI) retains endogenous stromal and immune components [159, 166]. In the ALI culture system, tumoroids are encapsulated in a collagen gel with one side exposed to air and the other side in contact with the liquid culture medium [161]. Based on the ALI culture method, LCOs and CRC organoids retain native CD45 + immune cells for more than 10 days but show a remarkable reduction in the CD3 + cell population [166]. Another study reported a modified ALI method to model the immune microenvironment of diverse malignancies [159]. This method enabled the presence and maintenance of immune cells, including NK cells, T cells, B cells, and macrophages, even during long-term culture for several months [159]. Generally, refined organoid technology through coculture systems may be a promising in vitro platform for modeling the tumor immune environment.

## Exploration of drug resistance-related mechanisms

Tumoroids reliably retain the pivotal characteristics of primary parent tumors, as described above [167], which facilitates the investigation of therapy resistance mechanisms. The study by Boos SL underlined the capacity of *KRAS* wild-type CRC organoids to simulate and reproduce gradual chemotherapy tolerance to the combination of irinotecan and EGFR inhibition in vitro [168]. In GCOs resistant to oxaliplatin, the presence of myoferlin was shown to be closely correlated with the acquisition of oxaliplatin resistance [80]. In another study, atypical cyclin P expression was revealed to promote stemness-like phenotypes of intestinal cancer organoids [169], which often led to tumor recurrence, metastasis, and therapy resistance. In addition, two tumoroids that both harbored *EGFR* p.L858R mutations responded differently to the EGFR inhibitor erlotinib [16]. Amplification of *MET* in the organoid was revealed to probably contribute to resistance to erlotinib, since the c-Met inhibitor crizotinib significantly suppressed the growth of erlotinib-resistant organoids [16].

Androgen receptor (AR) pathway inhibitors are among the clinically commonly applied drugs for the population with PC, exerting potent tumor-killing effects [170]. However, some patients with PC gradually become resistant to AR inhibitors, which might be explained by epigenetic reprogramming driving castration-resistant PC adenocarcinoma to neuroendocrine PC [170]. Researchers further confirmed that the ECM type differentially modulates the response of PC organoids to the epigenetic repressor EZH2 and dopamine receptor D2 [170]. This phenomenon suggests that ECM regulates the response to targeted therapies during the transition from castration-resistant PC adenocarcinoma to neuroendocrine PC [170]. The presence of MDSCs correlates with the resistance state of tumoroids with PD-L1 expression to nivolumab (a PD-1 receptor inhibitor) [156]. Collectively, these results indicate that organoids potentially represent an excellent tool for revealing drug-resistant mechanisms.

## Identification of novel tumor biomarkers

Tumoroids also exert great potential in discovering novel tumor biomarkers [4, 75, 100]. Ukai S and colleagues developed and harvested 5-FU-resistant GCOs [75]. A microarray analysis revealed that KH domain-containing, RNA binding, and signal transduction associated 3 represented an independent prognostic factor for patients with gastric cancer, especially for the population treated with 5-FU [75].

Another study documented the feasibility of employing PDAC PDX-derived organoids to identify promising and clinically significant biomarkers of extracellular vesicles for tumor detection and diagnosis with small amounts of media supernatant [4]. This study screened 241 proteins from 1465 identified proteins, focused on 5 markers (cluster of differentiation 44 (CD44), glypican 4, UGLUT2, CD14, and annexin A11), and identified annexin A11, CD44 variant 6, and glypican 4 as potential candidate biomarkers [4, 171].

## The potential for personalized medicine

### Predicting the drug response

Previous studies have witnessed consistency in determining drug responses among organoids, PDXs, and corresponding primary tumors from patients [13, 16, 24, 172, 173], indicating that organoids are a promising tool for predicting responses to therapeutic agents. PDOs established from metastatic gastrointestinal cancers serve as an excellent tool for predicting the response to targeted chemotherapy or novel agents with 93% specificity and 100% sensitivity [12]. The study by Seidlitz T verifies that GCOs sufficiently recapitulate divergent responses to chemotherapy, including 5-fluorouracil, irinotecan, oxaliplatin, docetaxel, and epirubicin, which are routinely applied drugs for gastric cancer [26]. The study by Grossman JE and colleagues revealed the concordance between the drug sensitivity of PDAC PDOs and the clinical responses of matched individual patients to the same antitumor drugs [96].

Wang Y has described the preliminary safety and antitumor efficacy of pyrotinib (a pan-HER receptor tyrosine kinase inhibitor) by studying tumoroids and corresponding xenografts derived from tumor samples from patients with HER2-A775_G776YVMA-inserted advanced lung adenocarcinoma [174]. Pyrotinib significantly inhibits the growth of organoids and induces a remarkable reduction in the tumor burden among mouse PDX models compared with afatinib [174]. Consistently, a phase II clinical trial enrolling a total of 15 patients with HER2-mutant NSCLC showed that patients benefited from pyrotinib treatment with an objective response rate of 53.3% and a median of approximately 6.4 months elapsed before tumor progression [174].

The PDO biorepository of locally advanced rectal cancer was generated by Yao Y and colleagues [112]. Patients whose tumor tissues were utilized to generate organoids in this study were enrolled in a phase III clinical trial. The results of this coclinical study showed that responses of rectal cancer organoids to chemoradiation displayed high similarity with the responses in matched patients with 84.43% accuracy [112]. Another study conducted by Ganesh K et al. revealed the heterogeneous responses of rectal cancer organoids to 5-FU treatment, FOLFOX (5-FU, leucovorin, and oxaliplatin) exposure and ionizing radiation, consistent with data from the corresponding patients [107]. The *KRAS*-mutant rectal cancer organoids showed resistance to the EGFR inhibitor cetuximab, whereas *KRAS*-wild-type tumoroids displayed sensitivity to this drug, consistent with the clinical trial showing that the *KRAS* mutation correlates with resistance to EGFR-targeted therapy [107].

Apart from chemotherapy and targeted therapy, tumoroids also show potential in predicting tumor responses to immunotherapies [175]. Using the ALI method, PDOs from NSCLC, melanoma, and renal cancer were successfully generated and contained various types of immune cells, including functional tumor-infiltrating lymphocytes, natural killer cells, and tumor‐associated macrophages [159]. Upon PD-1/PD-L1 blockade, activation of T cells and tumor killing activity were detected in 6 of 20 PDOs derived from immunotherapy‐responsive tumors, consistent with clinical trials of different tumor types [159]. According to the study by Votanopoulos, immune‐enhanced patient-derived tumoroids are successfully established through the incorporation of lymph nodes [175]. This study showed a high clinical correlation (85%) of these tumoroids with the response to checkpoint inhibitors [175]. Thus, refined PDOs may promote the prediction evaluation of individual tumor responses to clinically used immunotherapies.

Despite the great potential of organoids in predicting drug responses, the processes of tumoroid generation and drug testing are time-consuming, which hampers better clinical application of organoid technology. Encouragingly, a recent study reported successful one-week drug sensitivity testing in LCOs [22]. LCOs that were generated and analyzed using microwell arrays were applied to conduct drug sensitivity testing with the InSMAR-chip [22]. The one-week on-chip drug sensitivity faithfully recapitulated patients’ therapeutic responses, as verified by PDX models [22]. This short-term drug response testing of tumoroids using the InSMAR-chip makes organoids better suited for use in the clinic.

### Exploration of promising combination treatment strategies

Organoids have been shown to be a promising platform for exploring better combination treatment strategies. Previous studies based on tumoroids guided the selection of a combination treatment consisting of the KRAS-selective inhibitor AMG501 and EGFR inhibitor cetuximab for CRC organoids with *KRAS*^G12C^ mutation rather than single KRAS inhibitor exposure [113], the combination of the FGFR inhibitor BGJ398 and the mitogen-activated protein kinase inhibitor trametinib for FGFR1-aberrant NSCLC organoids instead of the combination of BGJ398 and PI3K inhibitor BKM120 [24], and combination treatment with MC3138 (a SIRT5 activator) and gemcitabine for patients with PDAC harboring low SIRT5 expression [176].

Mutational profiles of tumoroid lines may guide the selection of potentially effective combination chemotherapeutics. Based on the tumoroid platform, combination treatment consisting of EGFR pathway blockade and AURKA inhibition was found to be probably effective for chemoresistant CRC liver metastases with acquired *KRAS* mutation and increased expression of AURKA and c-MYC as a second-line treatment candidate [168]. Combination treatment with JNJ-42756493 (an FGFR inhibitor) plus AZD8055 or sirolimus (an mTOR inhibitor) exerted greater antitumor effects on organoids harboring *FGFR3* mutations and nonsense *TSC* mutations than single-drug treatment, which was validated in orthotopic PDXs [121].

Combination treatment with talazoparib (a PARP inhibitor) and CX-5461 (an inhibitor of RNA polymerase I transcription and an activator of the DNA damage response) might be a promising candidate treatment for HR-proficient patients who are not suitable for PARP inhibitor monotherapy, which was verified by increased DNA damage and decreased growth of organoids derived from HR-proficient castrate-resistant PC after treatment [177]. Based on tumoroids generated from circulating tumor cells of patients with HCC, oral cancer, or CRC, the *Antrodia cinnamomea* mycelium-derived bioactive compound GKB202 was indicated to be a promising adjuvant and enhancer for 5-FU-based treatment [178]. Collectively, organoids might serve as an excellent platform to explore novel and promising combination treatment strategies for patients with intractable tumor types.

### Discovery of novel anticancer targets and promising drug candidates

Organoids are also utilized to provide insights into possible therapeutic targets and facilitate the development of novel antitumor drugs, such as the SMAC mimetic LCL161 for liver metastatic rectal cancer organoids [114] and the novel CDK7 inhibitor YPN-005 for SCLC organoid lines [179]. A high-throughput screen based on the interaction of patient-derived BCOs and tumor-specific cytotoxic T cells identified three epigenetic inhibitors, BML-210, GSK-LSD1, and CUDC-101, with significant antitumor effects [180]. Additionally, BML-210 remarkably sensitized BC to a PD-1 inhibitor [180]. Based on the coculture of glioma organoids and human umbilical vein endothelial cells in fibrin gel, the drug atorvastatin dose-dependently exerts significant inhibitory effects on angiogenesis with downregulation of VEGF, CD31, and Bcl-2 [140]. This phenomenon indicated that atorvastatin might be a promising agent for glioblastoma treatment.

Both the tumor-suppressing or killing effects of drugs themselves and the efficient delivery of drugs into tumors are important for cancer treatment. Based on multicellular HCC organoids containing both HCC cells and diverse stromal cells (ECs, fibroblasts, and hepatic stellate cells) [181], high activity of Yes-associated protein/transcriptional coactivator with PDZ-binding motif signaling was shown to be associated with stromal activation in HCC and suppressed the penetration of verteporfin into tumoroids, indicating that treatments targeting activated cancer stroma might facilitate drug delivery into HCC [181].

## Tumor organoids versus PDX models

PDX models represent excellent in vivo imitations with more than 85% accuracy [182]. PDX models cannot be built before the direct transfer of a fresh biopsy specimen, tumor sample surgically removed from patients, malignant ascites-derived tumor cells, or circulating cancer cells into immunodeficient mice [183–185]. Tumor tissues contain tumor cells, tumor architectures, and a population of stromal cells [3]. Thus, PDX models can well recapitulate the histological characteristics, molecular features, and intertumoral and intratumor heterogeneity of parent tumors [186–188]. PDXs are often essential for authentication in clinical experiments [189] and have been extensively used for preclinical and translational cancer research [190–192].

Emerging evidence indicates that some limitations hamper its broad and extensive application in basic research and personalized medicine [193, 194]. First, these models are costly [195] and time-consuming [183, 193, 196]. Establishment of PDX models generally requires approximately 4–8 months for preclinical applications [5], which may lead to missing the best therapy opportunity for patients with cancer. Second, the stroma is not exactly the same as the original stromal components. After implantation, the original tumor-associated stroma derived from primary human tumors is gradually replaced by the extracellular matrix and fibroblasts of the hosts [197]. Third, ethical concerns and animal welfare should be considered. Fourth, the engraftment rate of these models remains low (average 30–40%) [193] and fluctuates substantially, depending on parent tumor types, malignancy grade [187, 197–199], implantation sites [5], and tumor tissue volume [5]. According to work from Yoko S DeRose, a higher success rate of PDX engraftment is significantly correlated with shorter overall survival of patients with breast cancer [187], indicating that a higher “take rate” of implantation is positively associated with a higher malignancy of primary tumors. In addition, the success rate of engraftment varies at different implantation sites. Common implantation sites include subcutaneous, subrenal capsule, and orthotopic sites. The identification of the best transplantation site is important. In addition, the volumes of some human tumors are originally small, such as head and neck squamous cell carcinoma and cholangiocarcinoma [5]. Sufficient tumor tissue is one of the prerequisites for PDX development. Insufficient tumor volume of these cancer types limits the generation of corresponding PDXs. Therefore, more efforts are needed to improve methods and techniques aimed at increasing the implantation efficiency of these tumor types.

PDXs and tumor organoids are both excellent platforms for basic research and translational medicine. However, some major differences have been noted between these two models. Compared with tumoroids, PDXs are in vivo models rather than in vitro models. PDX establishment requires a larger quantity of cells and more time and is more expensive. In addition, high-throughput analysis and screening of PDXs are more difficult, but PDXs can be more feasibly and better standardized. A comparison between PDXs and PDOs is shown in Table 2. After considering the advantages and disadvantages of these two models, one cannot be completely replaced by the other, but the combination of these two models might compensate for the shortcomings of the other. Researchers can develop appropriate options for distinct assays and experimental purposes.

**Table 2:** Comparison between patient-derived xenografts (PDXs) and patient-derived organoids (PDOs)

	PDXs	PDOs
Ex vivo, in vivo, or in vitro	In vivo	Ex vivo or in vitro
Use of immunodeficient animals	Yes	No
Quantity of cells for establishment	Large	Small
Establishment time	6–8 months	4–6 weeks
Initiation success	Moderate	Moderate
Cost	More expensive	Expensive
Genetic/epigenetic alterations	Similar	Similar
Pathohistological characteristics	Similar	Similar
Response to anticancer drugs	Similar	Similar
Reliability as preclinical models	Yes	Yes
Throughput	Low	Moderate
Standardization	Moderate	Low

## Limitations

Although organoids are honored as “approximating organs” and show great potential in basic cancer research and clinical applications, some current challenging bottlenecks and difficulties remain to be solved. First, organoid establishment, maintenance, and passages are costly. Second, the current success rates of establishing diverse cancer types vary substantially [121, 200]. A further improvement in the establishment rate is important and is affected by various factors, such as the cellularity of the corresponding primary tissues [12]. Third, optimized and standardized culture conditions for distinct tumoroids should be established to improve large-scale tumoroid reproducibility and facilitate the application of organoid technology in high-throughput drug screening. Fourth, the effects of current ECM components on tumoroid applications remain unclear. Fifth, tissue samples prepared for organoid generation are only small parts of the whole tumor. The higher heterogeneity of tumors questions the reliability of substituting small pieces for whole tumor tissues. Tissue extraction from different sites of the same tumors might better reflect tumor heterogeneity and reliably facilitate cancer translational research. Sixth, the current organoid technology is unable to easily replicate the complexity of the patient-specific immune environment. Although the coculture system of tumoroids and immune cells has promoted better modeling of tumor–immune interactions and their effects on treatment, some challenges might hamper the accurate modeling and prediction of responses to immunotherapies. For instance, different tumor types possess distinct immune components and different cell quantities, which affect the immune cell composition in the early stage of tumoroid culture and the option to maintain and expand these immune cells. Some tumors contain numerous and complex types of immune cells, while other tumor types only possess immune cells in the surrounding stroma or lack immune cells. The addition of HLA-mismatched allogeneic exogenous immune cells to organoid cultures might contribute to high background killing and reduce assay specificity [161]. In addition, although preserved immune cells can be maintained initially, they may be lost and diluted over time [201]. Imprecise modeling of the tumor immune environment prevents organoids from being useful for translational medicine and precision medicine. Seventh, vascularization of organoids is still a major challenge. Although implantation of organoids into animals or coculture systems promotes organoid vascularization, these methods only endow organoids with vascular characteristics but not functional perfusion vessels [189]. The current microfluidic platform used to establish vascularized organoids is crude and semiadjustable, and it is affected by multiple factors, including the concentration and composition of cytokines and flow rate. More accurate and flexibly controllable and detectable microfluidic platforms are urgently needed for better vascularization of organoids and accurate prediction of responses to antiangiogenic therapies.

## Conclusions

Organoids tailored to individual patients have revolutionized cancer research and show great potential in promoting personalized medicine. Tumoroids in proper culture systems faithfully retain morphological characteristics, genomic profiles, and mutational landscapes and recapitulate the genetic and phenotypic heterogeneity of the original tumors. Cocultures with stromal cells and immune components endow organoids with the capacity to model the TME. The accurate recapitulation of primary human tumors makes organoids an excellent platform for both basic research and translational medicine, including cancer modeling for the investigation of tumorigenesis and cancer progression, as well as drug response prediction, therapy optimization, and the discovery of novel antitumor drugs in a patient-specific manner. However, the current organoid system has some shortcomings, as described above, which hamper routine clinical application. More studies are needed to solve these problems and improve this technology.

## Abbreviations

AKT: Protein kinase B
ALI: Air–liquid interface
AR: Androgen receptor
BC: Breast cancer
BCOs: Breast cancer organoids
BME: Basement membrane extract
CAFs: Cancer-associated fibroblasts
CDK1: Cyclin-dependent kinase 1
CDX2: Caudal-related homeobox 2
CD44: Cluster of differentiation 44
CD90: Cluster of differentiation 90
CIN: Chromosomal instable
CK7: Cytokeratin 7
CRC: Colorectal cancer
3D: Three-dimensional
ECM: Extracellular matrix
ECs: Endothelial cells
EGF: Epidermal growth factor
*EGFR*: Epidermal growth factor receptor
ER: Estrogen receptor
FGF: Fibroblast growth factor
FGFR2: Fibroblast growth factor receptor 2
GCOs: Gastric cancer organoids
GS: Genomic stable
HCC: Hepatocellular carcinoma
HER2: Human epidermal growth receptor 2
iPSCs: Induced pluripotent stem cells
LC: Lung cancer
LCOs: Lung cancer organoids
MAPK: Mitogen-activated protein kinase
MCs: Mesenchymal cells
MDSCs: Myeloid-derived suppressor cells
MSI: Microsatellite instable
mTOR: Mammalian target of rapamycin
NSCLC: Non-small cell lung cancer
PC: Prostate cancer
PDAC: Pancreatic ductal adenocarcinoma
PD-L1: Programmed death-ligand 1
PDOs: Patient-derived organoids
PDX: Patient-derived xenograft
PI3K: Phosphatidylinositol 3-kinase
PR: Progesterone receptor
PSCs: Pluripotent stem cells
RCC: Renal cell carcinoma
TME: Tumor microenvironment
TGF-β: Transforming growth factor beta
VEGFR: Vascular endothelial growth factor receptor
